# Splenic Complications of *Babesia microti* Infection in Humans: A Systematic Review

**DOI:** 10.1155/2020/6934149

**Published:** 2020-05-27

**Authors:** Igor Dumic, Cristian Madrid, Libardo Rueda Prada, Charles W. Nordstrom, Pahnwat Tonya Taweesedt, Poornima Ramanan

**Affiliations:** ^1^Mayo Clinic Alix College of Medicine and Science, Rochester, MN, USA; ^2^Division of Hospital Medicine, Mayo Clinic Health System, Eau Claire, WI, USA; ^3^Icahn School of Medicine at Mount Sinai, New York City, NY, USA; ^4^Division of Infectious Disease, University of Colorado, Denver, CO, USA

## Abstract

Splenic complications of acute *Babesia microti* infection include splenomegaly, splenic infarct, and splenic rupture. These complications are relatively rarely reported, and the aim of this research was to synthetize data on this topic according to the Preferred Reporting Items for Systematic Reviews and Meta-Analyses (PRISMA) guidelines using the PubMed database. In this review, we find that unlike other severe complications of babesiosis, splenic infarct and rupture occur in younger and immunocompetent patients, and they do not correlate with parasitemia level. Furthermore, admission hemoglobin of 10 mg/dl or less, platelet count of 50 × 10⁹/L or less, presence of hemodynamic instability, and splenic rupture were associated independently with an increased risk of requiring splenectomy. As babesiosis is an emerging tick-borne zoonosis, we hope that this review will help to raise awareness among clinicians regarding this rare but potentially life-threatening complication.

## 1. Introduction

Babesiosis, also known as “Nantucket fever” or “malaria of the United States,” refers to an emerging vector-borne zoonosis caused by intraerythrocytic protozoans of the genus *Babesia* which infect and lyse red blood cells. In the US, babesiosis is endemic to the Pacific Northwest, Midwest, and the East Coast regions, particularly within the New England states [1, 2]. Its geographic distribution mimics that of Lyme disease, anaplasmosis, Powassan virus, and *Borreliamiyamotoi* infections, as all these pathogens are transmitted to humans through the same vector, *Ixodes scapularis* [1, 2]. The vast majority of cases in the US are caused by *B*. *microti*, with a smaller percentage of cases caused by *B*. *duncani*, found primarily in the NW US, and the recently reported *B*. *divergens-*like organisms [2, 3]. The first documented case in the US occurred in 1969, when an immunocompetent man from Massachusetts' Nantucket Island was diagnosed with *B*. *microti* infection (hence the name, “Nantucket fever”) [1, 4].

Babesiosis is transmitted mainly through bites from infected *I. scapularis* ticks; however, in rare cases, transmission may occur via the transplacental route, blood transfusion, or by organ transplant [5–8]. The incidence of tick-borne diseases in the US is increasing due to multiple factors including enlarging deer and tick populations, increased proximity between humans and ticks due to rural development, expanded awareness of tick-borne infections, availability of better diagnostic methods, and the effects of climate change [8, 9]. Climate change is predicted to have a significant impact on the incidence of tick-borne infections in endemic regions, with one study estimating a greater than 20% increase in the incidence of Lyme disease assuming a 2°C increase in the annual average temperature in the coming decades [10]. Clinical manifestations of babesiosis range from mild flu-like symptoms to life-threatening multiorgan failure with a fatality rate of 6–21% [11]. Asplenic individuals, the elderly, and immunocompromised patients have higher mortality rates due to the development of severe complications [11, 12]. Severe complications of *B*. *microti* infection include acute respiratory distress syndrome (ARDS), disseminated intravascular coagulation (DIC), and liver or renal failure [12–14]. Splenic rupture is a severe but rarely reported complication of *B. microti* infection. 

There have been few published case reports on this topic with literature review of varying extent, and no systematic review has been performed thus far to the best of our knowledge. In this systematic review, we have described the clinical features, laboratory findings, and the management of patients with splenic complications during the acute infection with *B*. *microti*. We have also reviewed the risk factors associated with splenic lesions in patients suffering from the disease. Subsequently, the review will help in raising awareness, among clinicians, on the potentially life-threatening splenic complications resulting from human babesiosis.

## 2. Materials and Methods

### 2.1. Database and the Key Words (MeSH)

A systematic review of the literature following the PRISMA guidelines (Preferred Reporting Items for Systematic Reviews and Meta-Analyses) was performed using the PubMed database for case reports and case series of *B*. *microti* infection with splenic complication from the date of database inception to September 2019. The following key words alone and/or in combination were used: “babesiosis AND splenic rupture,” “*Babesia microti* AND splenic rupture,” “babesiosis AND splenic infarct,” “*Babesia microti* AND splenic infarct,” “babesiosis AND splenic laceration,” and “*Babesia microti* AND splenic laceration.”

### 2.2. Definitions

We defined splenic complication as splenomegaly, splenic rupture, or splenic infarct depending on the radiological findings described in case reports. The presence of hemoperitoneum was indicative of splenic rupture, whereas splenic infarct was associated with intact splenic capsule and absence of hemoperitoneum [15]. Splenomegaly was defined as the spleen weighing more than 400 g and/or measuring greater than 11 cm in craniocaudal length. Massive splenomegaly was defined as the spleen weighing above 1000 g and/or measuring greater than 20 cm in craniocaudal length [16, 17]. Parasitemia was classified as severe if more than 10% of erythrocytes were infected on blood smear examination [18]. Diagnosis of babesiosis was considered delayed (misdiagnosed) if disease was not suspected on admission, inappropriate antibiotics were administered for up to 48 hours after admission, or if diagnosis was obtained postmortem.

### 2.3. Selection Criteria

**Figure 1:**
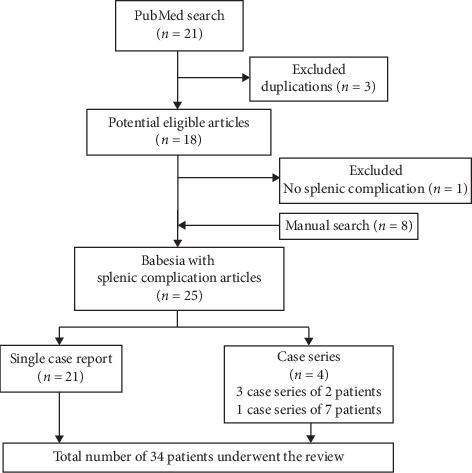
The flowchart delineates methodology and literature selection process according to PRISMA (Preferred Reporting Items for Systematic Reviews and Meta-Analyses).

We selected only definite cases of *B*. *microti* infection diagnosed by PCR, serology, and/or blood smear. We considered the blood smear alone diagnostic only in cases where the patient recovered following antibabesial therapy. Duplicate articles, articles in languages other than English, narrative review, and cases of babesiosis without splenic complication were excluded. The flowchart of selection of the final cases included in our analysis is illustrated in Figure 1.

### 2.4. Data Collection

Two researchers independently and blindly identified and selected the titles, abstracts, and full texts obtained in the database search. Discrepancies of the selected articles were resolved by consensus. After completing the PubMed PRISMA search, we completed a manual search by subsequently screening the reference lists of all selected articles. An Excel table was constructed, and for each case, we collected patient demographics, clinical presentation, medical comorbidities, vital signs on admission, time from presentation to diagnosis, type of splenic involvement, severity of parasitemia, patient's immune status, presence of coinfection, treatment, and clinical outcome (Table 1) [13, 19–41]. Of 34 cases reported, 19 cases were collected from single case reports; there were 4 case series, each including 2 cases [22, 23, 27, 37]. Finally, there was one case series including 7 patients from a single institution in Rhode Island, USA [39].

### 2.5. Statistical Analysis

The program Stata/MP 14.2 was used for statistical analysis. Patient characteristics are summarized using descriptive statistics; for example, medians and interquartile ranges (IQR) for continuous variables and counts and percentages for categorical variables. A multivariate regression model was used to assess the association of risk factors with splenectomy. Due to incomplete data in 4 cases, a total of 30 cases constituted the final sample. A *P* value of <0.05 was defined as statistically significant.

## 3. Results and Discussion

### 3.1. Demographic Data

The median patient age was 56 years (IQR 50–69.5). The youngest patient was 23, and the oldest was 85 years old. Of note, most other severe complications of babesiosis occurred primarily in the elderly [1, 2]. Curiously, while one recent study from a babesiosis-endemic area in the US reported a female predominance [14], we find that splenic complications occur almost exclusively in men. In our systematic review, 30 of 34 cases (88%) were males. Male predominance was also described in a recent case series (included in this systematic review) [13]; however, in that series, the median patient age was significantly lower (48 years). Furthermore, one recent retrospective study from Massachusetts showed that patients who developed splenic rupture were older than previously reported (median age 62) [42]. All reported cases in this review were from the US except for one patient who might have contracted the infection in Ecuador [33]. Another case was reported in South Korea; however, in that case, the disease had been acquired in New Jersey, USA [39]. In multivariate analysis, neither age nor sex was associated with splenectomy as an outcome (Table 2).

### 3.2. Comorbidities and Immune Status

Unlike other severe complications of *B*. *microti* infection (such as ARDS, DIC, acute renal injury, acute liver failure, and severe hemolytic anemia), splenic rupture does not appear to correlate with host immune status. None of the reported cases were immunocompromised (Table 1). Additionally, among cases that reported comorbidities, 14 out of 32 patients (43.75%) had none. The most common comorbidity was hypertension occurring in 7 out of 32 patients (21.87%), but in multivariate analysis, the presence of hypertension was not associated with splenectomy.

### 3.3. Clinical Manifestation and Coinfection

The most common presenting symptoms in this cohort of patients who developed splenic complications were as follows: fever (26/34, 76%), abdominal pain (20/34, 58%), chills and malaise (16/34, 47% for both), headache (7/34, 20%), night sweats (5/34, 14%), and syncope (3/34, 8.8%). It is interesting to note that 42% of patients with splenic complications of babesiosis did not complain of abdominal pain. This finding may contribute to the delay in diagnosis or misdiagnosis that was observed in 60% of patients (17/27, in 7 cases [13] was not reported). Unfortunately, coinfection with other tick-borne illness was not documented in many cases. Hence, we were unable to statistically examine if coinfection contributed to the development of splenic infarct and/or rupture, or if it was associated with an increased frequency of splenectomy.

### 3.4. Laboratory Findings

In this systematic review, the majority of patients had evidence of various degree of anemia and thrombocytopenia. Overall anemia was present in 94% of patients (32/34), and thrombocytopenia was present in 87% of patients (29/33). The median hemoglobin concentration was 9.4 mg/dl (IQR 8.5–10.7), and the median platelet count was 77 × 10⁹/L (IQR 53–99). On multivariate analysis, hemoglobin concentration <10 mg/dl and thrombocytopenia of 50 × 10⁹/L or below were significantly associated with increased probability of requiring a splenectomy. Levels of parasitemia were documented in 26 of 34 cases (76%) and ranged from 0.1 to 30% (median 1%). Parasitemia levels generally correlate with severity of illness in both babesiosis and malaria [1, 2, 43], with parasitemia level above 10% considered to be severe infection. Among those cases with reported parasitemia levels, it is interesting to note that only 11.5% (3/26) of patients developed severe parasitemia. In fact, nearly half (46%) the patients who developed splenic complications from babesiosis had parasitemia level which was ≤1%. This observation that splenic complications of babesiosis do not correlate with the severity of parasitemia was consistent with findings from previous reports [13, 32, 36]. The persistence of parasitemia and rapidity of clearance following initiation of therapy were not described in the majority of cases, prohibiting our ability to evaluate these variables in relation to the outcome of interest (splenectomy).

### 3.5. Types of Splenic Complication

The spleen is an essential organ for the clearance of intraerythrocytic parasites such as *Plasmodium* spp. and *Babesia* spp [44, 45]. Due to the lack of experimental data on the pathophysiology of splenic rupture in human babesiosis, theories are drawn based on data from studies of pathophysiology of malaria-related splenic complications [46–48].

Spleen size was documented in 26 patients. Of these, 23 had splenomegaly (88.5%) which is similar to findings from a recently published study that described it in 89% [42]. This is significantly higher than findings from a systematic review by Renzulli et al. [17] who found splenomegaly in 55% of patients with atraumatic splenic rupture. There was only one case of massive splenomegaly, and in 22 patients (95%), splenomegaly was mild. This is in contrast with malaria or viral infections such as EBV and CMV where splenic rupture is usually due to increased intracapsular pressure from massive splenomegaly. Splenic rupture (23 of 34 cases, 67.6%) was a more common complication of babesiosis than splenic infarct (11 of 34 cases, 32.4%). In multivariate analysis, development of splenic rupture was statistically significant with the increased probability of having a splenectomy.

### 3.6. Pathophysiology of Splenic Infarct and Rupture

Three main mechanisms by which *B*. *microti* might cause splenic complications have been proposed, and these draw parallels with studies on malaria-associated splenic rupture:

1. *B*. *microti* causes erythrocyte lysis by direct parasite invasion which leads to endothelial damage, formation of microthrombi, and release of vasoactive factors, resulting in localized necrosis of splenic tissue [34, 37].
2. Enhanced erythrocyte cytoadherence related to excessive proinflammatory cytokine production can cause mechanical obstruction of splenic microcirculation leading to infarction and rupture [47, 48]. Interestingly, this may occur even at low parasitemia levels, similar to infection with malaria [49].
3. The splenic macrophages are crucial in the clearance of infection, and in the process of “removal” of infected erythrocytes trapped in its venules, the spleen enlarges and subsequently may rupture. This final theory is supported by microscopic and macroscopic appearance of the ruptured spleens among patients infected with *P*. *vivax.* The spleen of these patients had significant red pulp hyperplasia with plasma cells, lymphocytes, immunoblasts, and large number of histiocytes exhibiting erythrophagocytic activity. On immunohistopathological analysis, these showed diffuse hypercellularity, massive proliferating plasma cells, and striking intrasinusoidal histiocytosis [50–52].

### 3.7. Treatment and Outcome

In this systematic review, 2 patients died from babesiosis which corresponded to a mortality rate of 5.8%. This is lower than the previously reported mortality rates of patients who developed malaria-related splenic rupture [53]. Importantly, both patients who died were older (75 and 85 years old), and one was coinfected with *Ehrlichia chaffeensis* which might have contributed to his mortality. Additionally, in both cases, diagnosis was delayed, and this emphasizes the importance of timely diagnosis to decrease morbidity and mortality. All patients received antimicrobials although many cases did not document the duration of therapy. Most patients with documented antimicrobial therapy were treated with azithromycin and atovaquone (21/26, 81%). Of note, the emergence of resistance to azithromycin and atovaquone (as defined by microbiological relapse after completing initial antimicrobial course and while on therapy) has been described in 3 immunocompromised patients who required prolonged treatment for recurrent relapses [54].

Transfusion requirements were documented in 21 cases. Of these, 12 patients (57%) required transfusion of PRBC, 1 patient required transfusion of platelets, and 1 patient required an exchange transfusion. The average number of PRBC units transfused per patient was 2.8. Since January 2011, when babesiosis became a reportable disease, transfusion-transmitted babesiosis cases have sharply increased, and it is now the most common transfusion-transmitted pathogen [7, 55]. Since asplenic patients tend to have more severe disease, it is important to recognize the need to test patients for babesiosis if postsplenectomy fever develops. Furthermore, splenic rupture might be the first manifestation of babesiosis, and splenectomy can increase the severity of the disease [23, 36]. Of the 32 cases that survived which were described in this systematic review, 10 patients underwent splenectomy (31.2%) while 22 (68.8%) were managed conservatively with close observation, pain control, transfusion, hydration, and spleen preservation. In 12.5% of cases, splenic artery embolization was done to control the bleeding. Conservative management is preferable to avoid postoperative complications and complications from asplenia. Given the fact that all patients who develop splenic complications of babesiosis either live in or frequently visit *Babesia* endemic regions, splenic preservation is of utmost importance to decrease mortality in case of subsequent infections.

Our study has few limitations. We reviewed the cases that were only published in journals that are indexed in the PubMed database. Additionally, we have not reviewed cases published in languages other than English which contributes to publication bias.

## 4. Conclusion

This systematic review highlights the clinical presentation and outcomes of patients presenting with splenic complications of babesiosis. We note that splenic complications of babesiosis (unlike other severe complications of this infection) do not correlate with advanced patient age, host immunosuppression, or severity of parasitemia. Nearly half of the patients with splenic complications of babesiosis did not endorse abdominal pain which probably contributed to the delay in diagnosis or misdiagnosis that was observed in more than 60% of patients. In multivariate analysis, admission hemoglobin of 10 mg/dl or less, platelet count of 50 × 10⁹/L or less , presence of hemodynamic instability, and splenic rupture were associated with an increased risk of requiring splenectomy.

## Figures and Tables

**Table 1:** Demographic data, clinical features, management, and outcome of patients who developed splenic complications during acute Babesia microti infection.

Case (ref)	Year (author)	Age	Sex	US state, country	Comorbid conditions	Symptoms duration PTP (days)	Dx missed initially	Remembers tick bite	SIRS/HD unstable on admission	Hgb (md/dl)	Platelet (×10^9^/L)	Coinfection	Parasitemia level (%)	Splenomegaly	Type of splenic involvement PTA or AA	Transfusion (units)	ICS	Antibiotic treatment	Splenectomy
1 [19]	2001 (Javed)	85	M	NJ, USA	HTN	Fever, chills, malaise (7–14)	Yes	No	No	12	23	Yes *E.ch.*	8	No	I	Yes (2)	No	TCV + CLIN + QI	No, expired
2 [20]	2008 (Kuwaya ma)	61	M	NJ, USA	None	Fever, chills, malaise, AP (180) intermittent	Yes	No (golfing)	No	11	33	No	5	Yes	R (AA)	Yes (4)	No	AZ + ATQ (7)	Yes
3 [21]	2008 (Froberg)	56	M	MN, USA	HTN	Night sweats, malaise (NR)	Yes	NR	Yes	9.3	37	Yes (LD)	NR	Yes	R (AA)	NR	No	NR	Yes
4 [22]	2008 (Florescu)	58	M	NJ, USA	None	Fever, AP, malaise, syncope (5)	Yes	No	No	13.6	209	APG	0.5	Yes	I		No	AZ + ATQ (NR)	No
5 [22]	2008 (Florescu)	75	F	NY, USA	HTN	Night sweats, malaise (NR)	Yes	No	Yes	8.7	55	No	NR	Yes (massive)	I	NR	No	AZ + ATQ QI + CLIN	No, expired
6 [23]	2008 (Sidertis)	50	M	NJ, USA	None	Fever, AP, malaise, syncope (5)	Yes	No (golfing)	Yes	9.3	458	NR	3	Yes	R (PTA)	Yes (2)	No	AZ + ATQ (NR)	Yes
7 [23]	2008 (Sidertis)	71	M	NJ, USA	NR	Syncope (NR)	No	NR	Yes	9.2	72	NR	NR	Yes	R (PTA)	Yes (2)	No	CLIN + QI (10)	Yes
8 [24]	2011 (Tobler)	54	M	MA, USA	None	Nausea, chills, malaise, fever, AP (2)	No	No	No	10.2	26	No	3	Yes	R (PTA)	Yes (platelet)	No	AZ + ATQ (10)	No
9 [25]	2011 (Abbas)	23	M	CT, USA	None	Fever, chills, weight loss, malaise LDN (0.5)	Yes	Yes	No	6.8	78	No	30	Yes	R (PTA)	Yes (4)	No	CLIN + QI (10)	No
10 [26]	2011 (Reis)	70	M	NY, USA	NR	Fever, nausea, vomiting (3)	No	No	No	8.5	90	No	NR	Yes	R (PTA)	Yes (2)	No	AZ + ATQ (14)	No, SA embolization
11 [27]	2011 (El Khouri)	70	M	NY, USA	None	Fever, chills, malaise (4)	No	NR	No	10.1	77	No	8	Yes	R (PTA)	Yes (3)	No	CLIN + QI AZ + ATQ (16)	No, SA embolization
12 [27]	2011 (El Khouri)	36	M	NY, USA	None	Fever, headache, chills, cough (14)	No	NR	No	8.5	133	No	3.5	Yes	R (AA)	No	No	AZ + ATQ (21)	No
13 [28]	2011 (Wormser)	55	M	RI, USA	Diverticulosis	Fever, rash, AP (10)	Yes	NR	Yes	13.4	99	NR	0.1	NR	R (PTA)	NR	No	AZ + ATQ (42) relapse after 10-day course	Yes
14 [29]	2013 (Seible)	83	M	MA, USA	NR	Fever, nausea, AP (2)	No	NR	NR	NR	66	No	14	NR	R (PTA)	Yes exchange	No	CLIN + QI (30)	No
15 [30]	2013 (Leinwan d)	48	M	CT, USA	None	Headache, AP, malaise (7)	No	No (camping)	No	10.5	137	No	10	Yes	R (PTA)	NR	No	AZ + ATQ (NS)	No observation
16 [31]	2014 (Usatti)	54	M	CT, USA	None	Fever, headache, AP (2)	Yes	NR	Yes	10.6	123	No	NR	Yes	R (PTA)	Yes (2)	No	AZ + ATQ (10)	No
17 [32]	2015 (Farber)	59	F	CT, USA	HLP depression	Fever, chills, syncope, fatigue, AP (14)	No	No (gardening)	Yes	8.2	127	No	1	Yes	R (PTA)	Yes (2)	No	CLIN + AZ + ATQ (14)	Yes
18 [33]	2016 (Al Zoubi)	72	M	Ecuador	HTN	Fever, chills, weight loss, AP (NR)	Yes	NR	No	7.8	55	No	0.5	Yes	I	NR	No	ATQ + PRG (NR)	No
19 [34]	2016 (Wong)	56	M	NY, USA	Diabetes	Fever, malaise, night sweats (6)	Yes	No (landscaping)	No	10.7	163	Yes (LD)	1.5	No	I	No	NO	AZ + ATQ (10)	No SA embolization
20 [35]	2017 (Permpalu ng)	59	M	MA USA	MVP	Headache, fever, AP (14)	Yes	NR	NR	10.4	156	No	NR	Yes	I	No	No	AZ + ATQ (10)	No SA embolization
21 [36]	2018 (Dumic)	79	F	WI, USA	HTN atrial fibrillation CAD	Chest pain, dizziness (1)	Yes	Yes	Yes	6.5	6.5	Yes (LD)	1.3	No	R (PTA)	Yes (4)	No	AZ + ATQ (10) Doxy (21)	Yes
22 [37]	2018 (Blackwo od)	51	M	RI, USA	HTN atrial fibrillation	Fever, chills, malaise, AP (5)	No	Yes	Yes	9.3	25	NR	0.25	Yes	R (PTA)	Yes (4)	No	AZ + ATQ (14)	Yes
23 [37]	2018 (Blackwo od)	61	M	RI, USA	HTN, HLP	Fever, chills, malaise, AP (3)	Yes	No (gardening)	Yes	12.4	89	NR	0.44	Yes	I	No	No	AZ + ATQ (NR)	No
24 [38]	2018 (Li)	48	M	RI, USA	Asthma	Fever, chills, night sweats, AP (7)	NR	NR	No	8.8	68	NR	0.22	YES	R (NR)	NR	No	NR	No SA embolization
25 [13]	2018 (Patel)	48	M	RI, USA	Asthma	Fever, chills, night sweats, AP (7)	NR	NR	No	8.8	68	NR	0.22	Yes	R (NR)	NR	No	NR	No SA embolization
26 [13]	2018 (Patel)	83	M	RI, USA	CKD COPD	AP (1)	NR	NR	Yes	11.3	94	NR	0.30	NR	R (NR)	NR	No	NR	No
27 [13]	2018 (Patel)	40	M	RI USA	Diabetes	Fever, malaise, night sweats (5)	NR	NR	No	7.8	74	NR	0.07	NR	R (NR)	NR	No	NR	No
28 [13]	2018 (Patel)	51	M	RI, USA	HTN atrial fibrillation	Night sweats, headache, malaise, AP (3)	NR	NR	Yes	8.7	25	NR	0.25	18.2	R (NR)	NR	No	NR	Yes
29 [13]	2018 (Patel)	48	M	RI, USA	None	Fatigue, AP (5)	NR	NR	Yes	7.9	59	NR	0.26	16	R (NR)	NR	No	NR	Yes
30 [13]	2018 (Patel)	36	M	RI, USA	None	Fever, AP (4)	NR	NR	No	10.6	80	NR	0.35	NR	R (NR)	NR	No	NR	No
31 [13]	2018 (Patel)	68	M	RI, USA	HTN	Malaise, AP (NR)	NR	NR	No	6.6	NR	NR	5	NR	R (NR)	NR	No	NR	No
32 [39]	2018 (Kwon)	50	F	South Korea (imported from NJ, USA)	None	Headache, fever, AP, chills (NR)	Yes	NR (gardening)	No	11.2	53	NR	NR	NR	I	NR	No	ATQ/+AZ (NR)	No
33 [40]	2019 (Alvi)	60	M	CT, USA	HTN, diabetes, HLP	Fever, chills, rigors, AP (7–10)	Yes	No	Yes	10.2	36	No	11	Yes	I	Yes	No	NS AZ + ATQ (14)	No observation
34 [41]	2019 (Gupta)	53	M	NY, USA	None	Fever, chills, weakness (7)	No	No (hiking)	NR	11.2	90	NR	1.5	NR	I	No	No	AZ + ATQ (NS)	No observation

A: azithromycin; AA: after admission; AP: abdominal pain; APG: *Anaplasma phagocytophilum*; ATQ: atovaquone; CKD: chronic kidney disease; CLIN: clindamycin; CT: Connecticut; Dx: diagnosis; *E. ch*: *Ehrlichia chaffeensis*; F: female; HA: headache; HD: hemodynamic; Hgb: hemoglobin, HLP: hyperlipidemia; HTN: hypertension; I: infarction; ICS immunocompromised state; LD: Lyme disease; LDN: lymphadenopathy; M: male; MA: Massachusetts; MN: Minnesota; MVP mitral valve prolapse; NJ: New Jersey; NR: not reported; NS: not specified; NY: New York; PRG: proguanil; PTA: prior to admission; PTP: prior to presentation; QI: quinine; R: rupture; Ref: reference; SA: splenic artery; TCV: ticarcillin-clavulanic acid “SIRS: systemic inflammatory response syndrome”.

**Table 2:** Estimates of the linear probability model (LPM) for splenectomy.

Dependent variable: splenectomy	LPM
Age ≥55 years	0.2717^*∗*^ (0.1539)
Male	−0.0540 (0.1395)
Hypertension	−0.1861 (0.1419)
Hemoglobin <10 mg/dl	0.2795^*∗∗*^ (0.1178)
Platelets < 50 × 10^9^	0.3876^*∗∗*^ (0.1493)
Spleen rupture	0.4359^*∗∗*^ (0.1579)
Hemodynamic instability	0.4721^*∗∗∗*^ (0.1621)
Number of cases	30
R-squared	0.6752

This table reports the estimates of a linear probability model (LMP) for the binary variable splenectomy. The *R*-squared value of 0.6752 indicates that 67.52% of the variance in the dependent variable (splenectomy) is explained by the regression model. It also has the simple interpretation that it equals the difference between the average predicted probability in the two groups. Because of the well-known heteroskedasticity in the LPM, heteroskedasticity-robust standard errors are reported in parentheses. ^*∗∗∗*^*p* value <0.01, ^*∗∗*^*p* value <0.05, and ^*∗*^*p* value <0.1.

## References

[1] Vannier E. G., Diuk-Wasser M. A., Ben Mamoun C., Krause P. J. (2015). Babesiosis. *Infectious Disease Clinics of North America*.

[2] Vannier E., Krause P. J. (2012). Human babesiosis. *New England Journal of Medicine*.

[3] Burgess M. J., Rosenbaum E. R., Pritt B. S. (2017). Possible transfusion-transmitted babesia divergens-like/mo-1 infection in an arkansas patient. *Clinical Infectious Diseases*.

[4] Western K. A., Benson G. D., Gleason N. N., Healy G. R., Schultz M. G. (1970). Babesiosis in a massachusetts resident. *New England Journal of Medicine*.

[5] Joseph J. T., Purtill K., Wong S. J. (2012). Vertical transmission of *Babesia microti*, United States. *Emerging Infectious Diseases*.

[6] Cornett J. K., Malhotra A., Hart D. (2012). Vertical transmission of babesiosis from a pregnant, splenectomized mother to her neonate. *Infectious Diseases in Clinical Practice*.

[7] Leiby D. A. (2011). Transfusion-transmitted babesia spp.: bull’s-eye on *Babesia microti*. *Clinical Microbiology Reviews*.

[8] Brennan M. B., Herwaldt B. L., Kazmierczak J. J. (2016). Transmission of *Babesia microti* parasites by solid organ transplantation. *Emerging Infectious Diseases*.

[9] Knapp K. L., Rice N. A. (2015). Human coinfection with *Borrelia burgdorferi* ansd *Babesia microti* in the United States. *J Parasitol Res*.

[10] Dumic I., Severnini E. (2018). “Ticking bomb”: the impact of climate change on the incidence of lyme disease. *Canadian Journal of Infectious Diseases and Medical Microbiology*.

[11] Hatcher J. C., Greenberg P. D., Antique J., Jimenez-Lucho V. E. (2001). Severe babesiosis in long Island: review of 34 cases and their complications. *Clinical Infectious Diseases*.

[12] Krause P. J., Gewurz B. E., Hill D. (2008). Persistent and relapsing babesiosis in immunocompromised patients. *Clinical Infectious Diseases*.

[13] Patel K. M., Johnson J. E., Reece R., Mermel L. A. (2019). Babesiosis-associated splenic rupture: case series from a hyperendemic region. *Clinical Infectious Diseases*.

[14] Fida M., Douglas C., Ahmed H., O’horo J., Abu Saleh O. (2019). Babesiosis: a retrospective review of 38 cases in the upper midwest. *Open Forum Infectious Diseases*.

[15] Unal E., Onur M. R., Akpinar E. (2016). Imaging findings of splenic emergencies: a pictorial review. *Insights into Imaging*.

[16] Chapman J., Azevedo A. M. (2019). *Splenomegaly*.

[17] Renzulli P., Hostettler A., Schoepfer A. M., Gloor B., Candinas D. (2009). Systematic review of atraumatic splenic rupture. *British Journal of Surgery*.

[18] Homer M. J., Aguilar-Delfin I., Telford S. R., Krause P. J., Persing D. H. (2000). Babesiosis. *Clinical Microbiology Reviews*.

[19] Javed M. Z., Srivastava M., Zhang S., Kandathil M. (2001). Concurrent babesiosis and ehrlichiosis in an elderly host. *Mayo Clinic Proceedings*.

[20] Kuwayama D. P., Briones R. J. (2008). Spontaneous splenic rupture caused by *Babesia microti* Infection. *Clinical Infectious Diseases*.

[21] Froberg M. K., Dannen D., Bernier N., Shieh W. J., Guarner J., Zaki S. (2008). Case report: spontaneous splenic rupture during acute parasitemia of *Babesia microti*. *Annals of Clinical & Laboratory Science*.

[22] Florescu D., Sordillo P. P., Glyptis A. (2008). Splenic infarction in human babesiosis: two cases and discussion. *Clinical Infectious Diseases*.

[23] Siderits R., Mikhail N., Ricart C., Abello-Poblete M. V., Wilcox C., Godyn J. J. (2008). Babesiosis, significance of spleen function illustrated by postsplenectomy course in 3 cases. *Infectious Diseases in Clinical Practice*.

[24] Tobler W. D., Cotton D., Lepore T. (2011). Case report: successful non-operative management of spontaneous splenic rupture in a patient with babesiosis. *World Journal of Emergency Surgery*.

[25] Abbas H. M., Brenes R. A., Ajemian M. S. (2011). Successful conservative treatment of spontaneous splenic rupture secondary to babesiosis: a case report and literature review. *Connecticut Medicine*.

[26] Reis S. P., Maddineni S., Rozenblit G., Allen D. (2011). Spontaneous splenic rupture secondary to *Babesia microti* infection: treatment with splenic artery embolization. *Journal of Vascular and Interventional Radiology*.

[27] El Khoury M. Y., Gandhi R., Dandache P., Lombardo G., Wormser G. P. (2011). Non-surgical management of spontaneous splenic rupture due to *Babesia microti* infection. *Ticks and Tick-Borne Diseases*.

[28] Wormser G. P., Lombardo G., Silverblatt F. (2011). Babesiosis as a cause of fever in patients undergoing a splenectomy. *The American Surgeon*.

[29] Seible D. M., Khatana S. A. M., Solomon M. P., Parr J. B. (2013). Hoof beats may mean zebras: atraumatic splenic rupture. *The American Journal of Medicine*.

[30] Leinwand J. C., Arroyo J. P., Solomon D., Kaplan L. J. (2013). *Babesia microti* infection presenting as acute splenic laceration. *Surgical Infections*.

[31] Usatii N., Khachatrian A., Stratidis J. (2014). Spontaneous splenic rupture due to *Babesia microti* infection: case report and review of the literature. *IDCases*.

[32] Farber F. R., Muehlenbachs A., Robey T. E. (2015). Atraumatic splenic rupture from babesia: a disease of the otherwise healthy patient. *Ticks and Tick-Borne Diseases*.

[33] Al Zoubi M., Kwak T., Patel J., Kulkarni M., Kallal C. A. (2016). Atypical challenging and first case report of babesiosis in Ecuador. *IDCases*.

[34] Wong D. (2016). Babesia parasitemia causing splenic infarction: a review of the literature. *Case Reports in Internal Medicine*.

[35] Permpalung N., Valdivia L., Seshadri P., Rowley C. F. (2017). Acute atraumatic splenic hemorrhage: babesiosis or acute infectious mononucleosis. *The American Journal of Medicine*.

[36] Dumic I., Patel J., Hart M., Niendorf E. R., Martin S., Ramanan P. (2018). Splenic rupture as the first manifestation of *Babesia microti* infection: report of a case and review of literature. *American Journal of Case Reports*.

[37] Blackwood B., Binder W. (2018). Unusual complications from Babesia infection: splenic infarction and splenic rupture in two separate patients. *The Journal of Emergency Medicine*.

[38] Li S., Goyal B., Cooper J. D., Abdelbaki A., Gupta N., Kumar Y. (2018). Splenic rupture from babesiosis, an emerging concern? a systematic review of current literature. *Ticks and Tick-Borne Diseases*.

[39] Kwon H. Y., Im J. H., Park Y.-K., Durey A., Lee J.-S., Baek J. H. (2018). Two imported cases of babesiosis with complication or co-infection with lyme disease in republic of Korea. *The Korean Journal of Parasitology*.

[40] Alvi A., Gupta S., Goyal P., Pichardo J., Mattana J. (2019). Splenic infarction as a rare presentation of severe babesiosis. *IDCases*.

[41] Gupta A., Patel P., Manvar K., Kellner T., Guevara E. (2019). Splenic infarction in babesiosis: a rare presentation. *Clinical Case Reports*.

[42] Mojtahed A., Bates D. D. B., Hahn P. F. (2020). Splenic findings in patients with acute babesiosis. *Abdominal Radiology*.

[43] Trampuz A., Jereb M., Muzlovic I., Prabhu R. M. (2003). Clinical review: severe malaria. *Critical Care*.

[44] Djokic V., Akoolo L., Parveen N. (2018). *Babesia microti* infection changes host spleen architecture and is cleared by a th1 immune response. *Frontiers in Microbiology*.

[45] Djokic V., Primus S., Akoolo L., Chakraborti M., Parveen N. (2018). Age-related differential stimulation of immune response by *Babesia microti* and *Borrelia burgdorferi* during acute phase of infection affects disease severity. *Frontiers in Immunology*.

[46] Hwang J.-H., Lee C.-S. (2014). Malaria-induced splenic infarction. *The American Journal of Tropical Medicine and Hygiene*.

[47] Hemmer R. M., Ferrick D. A., Conrad P. A. (2000). Role of t cells and cytokines in fatal and resolving experimental babesiosis: protection in TNFRp55 −/− mice infected with the human babesia wa1 parasite. *The Journal of Parasitology*.

[48] Krause P. J., Daily J., Telford S. R., Vannier E., Lantos P., Spielman A. (2007). Shared features in the pathobiology of babesiosis and malaria. *Trends in Parasitology*.

[49] Bonnard P., Guiard-Schmid J.-B., Develoux M., Rozenbaum W., Pialoux G. (2005). Splenic infarction during acute malaria. *Transactions of the Royal Society of Tropical Medicine and Hygiene*.

[50] Machado Siqueira A., Lopes Magalhães B. M., Cardoso Melo G. (2012). Spleen rupture in a case of untreated *Plasmodium vivax* infection. *PLoS Neglected Tropical Diseases*.

[51] Kim K. M., Bae B. K., Lee S. B. (2014). Spontaneous splenic rupture in *Plasmodium vivax* malaria. *Annals of Surgical Treatment and Research*.

[52] Buffet P. A., Safeukui I., Deplaine G. (2011). The pathogenesis of *Plasmodium falciparum* malaria in humans: insights from splenic physiology. *Blood*.

[53] Imbert P., Rapp C., Buffet P. A. (2009). Pathological rupture of the spleen in malaria: analysis of 55 cases (1958–2008). *Travel Medicine and Infectious Disease*.

[54] Wormser G. P., Prasad A., Neuhaus E. (2010). Emergence of resistance to azithromycin-atovaquone in immunocompromised patients with *Babesia microti* infection. *Clinical Infectious Diseases*.

[55] Levin A. E., Krause P. J. (2016). Transfusion-transmitted babesiosis. *Current Opinion in Hematology*.

